# Gear and survey efficiency of patent tongs for oyster populations on restoration reefs

**DOI:** 10.1371/journal.pone.0196725

**Published:** 2018-05-02

**Authors:** David M. Schulte, Romuald N. Lipcius, Russell P. Burke

**Affiliations:** 1 US Army Corps of Engineers, Norfolk District, Norfolk, Virginia, United States of America; 2 Virginia Institute of Marine Science, College of William & Mary, Gloucester Point, Virginia, United States of America; 3 Department of Organismal and Environmental Biology, Christopher Newport University, Newport News, Virginia, United States of America; Bigelow Laboratory for Ocean Sciences, UNITED STATES

## Abstract

Surveys of restored oyster reefs need to produce accurate population estimates to assess the efficacy of restoration. Due to the complex structure of subtidal oyster reefs, one effective and efficient means to sample is by patent tongs, rather than SCUBA, dredges, or bottom cores. Restored reefs vary in relief and oyster density, either of which could affect survey efficiency. This study is the first to evaluate gear (the first full grab) and survey (which includes selecting a specific half portion of the first grab for further processing) efficiencies of hand-operated patent tongs as a function of reef height and oyster density on subtidal restoration reefs. In the Great Wicomico River, a tributary of lower Chesapeake Bay, restored reefs of high- and low-relief (25–45 cm, and 8–12 cm, respectively) were constructed throughout the river as the first large-scale oyster sanctuary reef restoration effort (sanctuary acreage > 20 ha at one site) in Chesapeake Bay. We designed a metal frame to guide a non-hydraulic mechanical patent tong repeatedly into the same plot on a restored reef until all oysters within the grab area were captured. Full capture was verified by an underwater remotely-operated vehicle. Samples (n = 19) were taken on nine different reefs, including five low- (n = 8) and four high-relief reefs (n = 11), over a two-year period. The gear efficiency of the patent tong was estimated to be 76% (± 5% standard error), whereas survey efficiency increased to 81% (± 10%) due to processing. Neither efficiency differed significantly between young-of-the-year oysters (spat) and adults, high- and low-relief reefs, or years. As this type of patent tong is a common and cost-effective tool to evaluate oyster restoration projects as well as population density on fished habitat, knowing the gear and survey efficiencies allows for accurate and precise population estimates.

## Introduction

To manage the oyster fishery effectively or monitor the performance of oyster populations on subtidal sanctuary reefs, accurate and precise population data, as well as data on the status of the shell or other materials the reef is constructed of are needed, taken by reliable gear with sufficient samples to control for variation over the reefs [1, 2]. Of the available methods to survey subtidal oyster populations, an effective one is SCUBA [3–6] when divers bring the sample to the surface, where it is processed on the vessel or in a laboratory. However, the cost to obtain statistically sufficient numbers of samples, usually 20 or more per reef can be prohibitively expensive. Non-destructive means of surveying oyster habitat include towed underwater video [5] and remotely-operated vehicle (ROV) video [7], as well as visual inspection by divers. Visual methods can provide information on the general condition of oyster habitat, but the means to accurately estimate oyster demographics from video have not been fully developed. Another effective method to physically sample subtidal oyster populations and habitat efficiently is to adapt oyster fishing gear to this purpose [7–9].

The two main choices in gear type are dredge and tong. These two gears are the preferred methods to harvest oysters in the commercial fishery, and have been designed to gather oysters efficiently from their reef habitat. Both dredges and tongs were used in some of the earliest surveys in Chesapeake Bay [10, 11], with tongs being used in shallow water and dredges in deeper waters. Estimates derived from dredges are not accurate due to problems relating to the efficiency of the device [10]. Since these early efforts, more recent studies [3, 12–15] have noted that dredge efficiency is low, highly variable and generally unreliable, though in some cases a dredge may be calibrated to provide a relative measure of population status [14].

**Fig 1:**
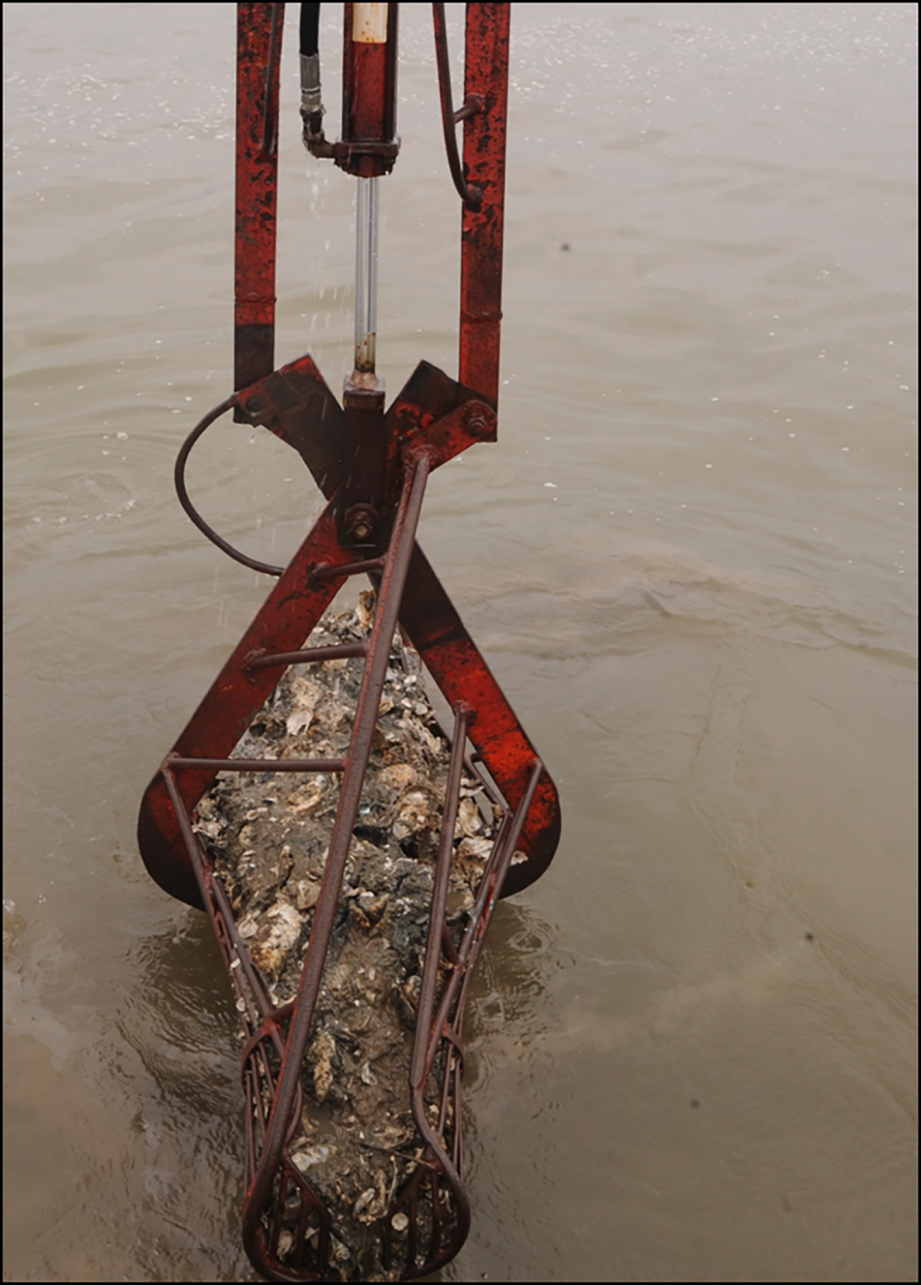
Photograph of a hydraulic patent tong.

**Fig 2:**
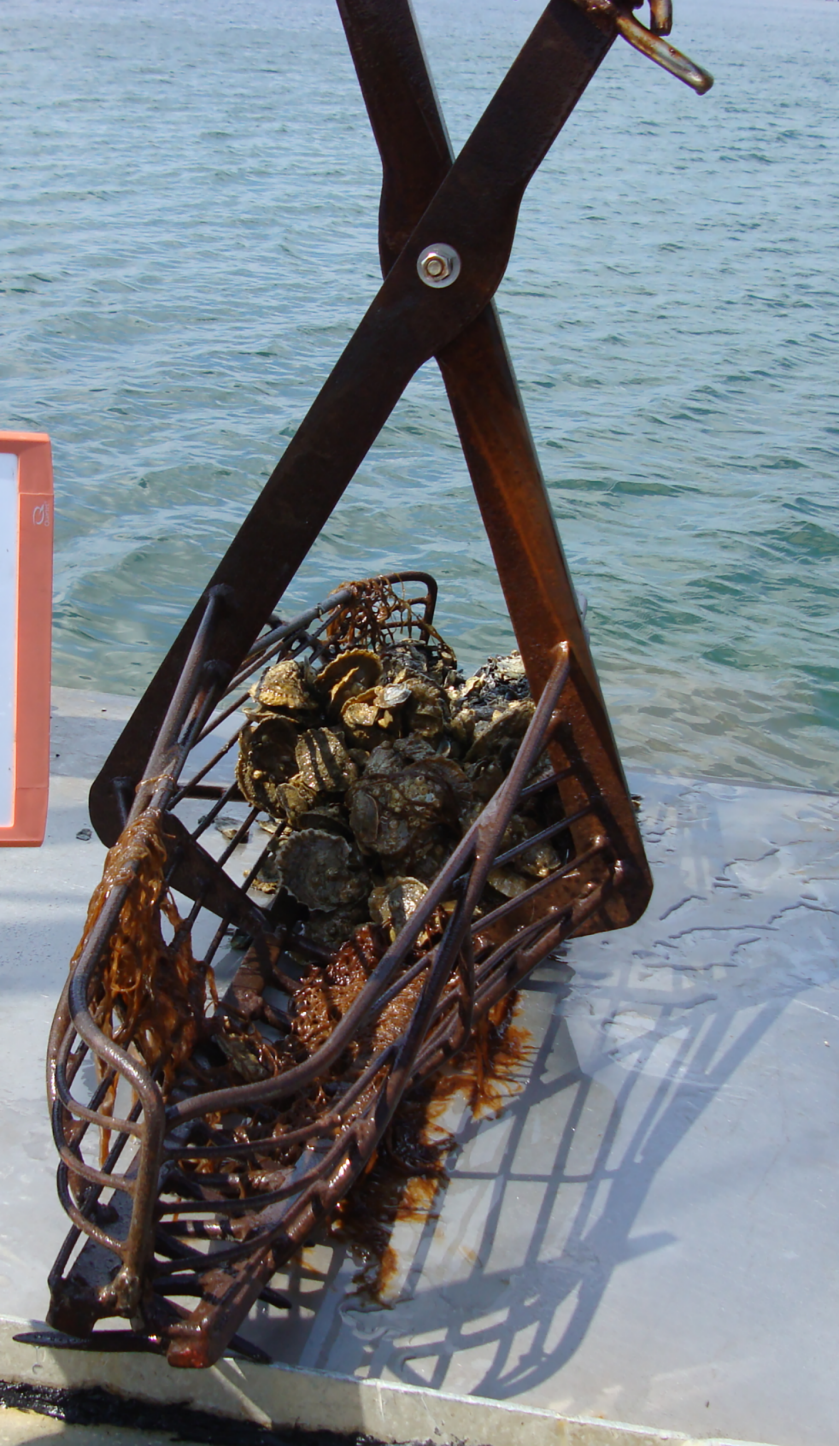
Photograph of a mechanically-operated patent tong.

There are two types of patent tongs, hydraulic and mechanical. Hydraulic tongs (Fig 1) are closed using hydraulic power, and are heavier than mechanically-operated tongs (Fig 2). Mechanical tongs are held open by a spring-loaded device, which is triggered and releases when the tong hits the bottom. The tong then closes as it is retrieved. When compared to divers, hydraulic patent tongs are assumed to be similar in efficiency to SCUBA divers, which are thought to be 100% efficient [3], but this has not been tested. Divers use digging implements and collect all oysters, live and dead, as well as all shell material down to 10 cm depth from a sample area. Similarly, a hydraulic patent tong collects samples down to 10 cm in depth. Hydraulic patent tongs require a larger vessel with specialized equipment. Instead, monitoring programs often use either the more commonly available dredges or mechanically-operated patent tongs whose efficiency has never been quantified. During a survey, only a single grab of the tong per sample site is taken, so it is important to determine the efficiency rate of this grab vs the total number of oysters present within the grab area, as we do not expect the tong to capture all oysters within a grab area on the first grab. This is the efficiency of the gear; when a sub-sample of this first grab is taken and processed, we consider this “survey efficiency.”

**Fig 3:**
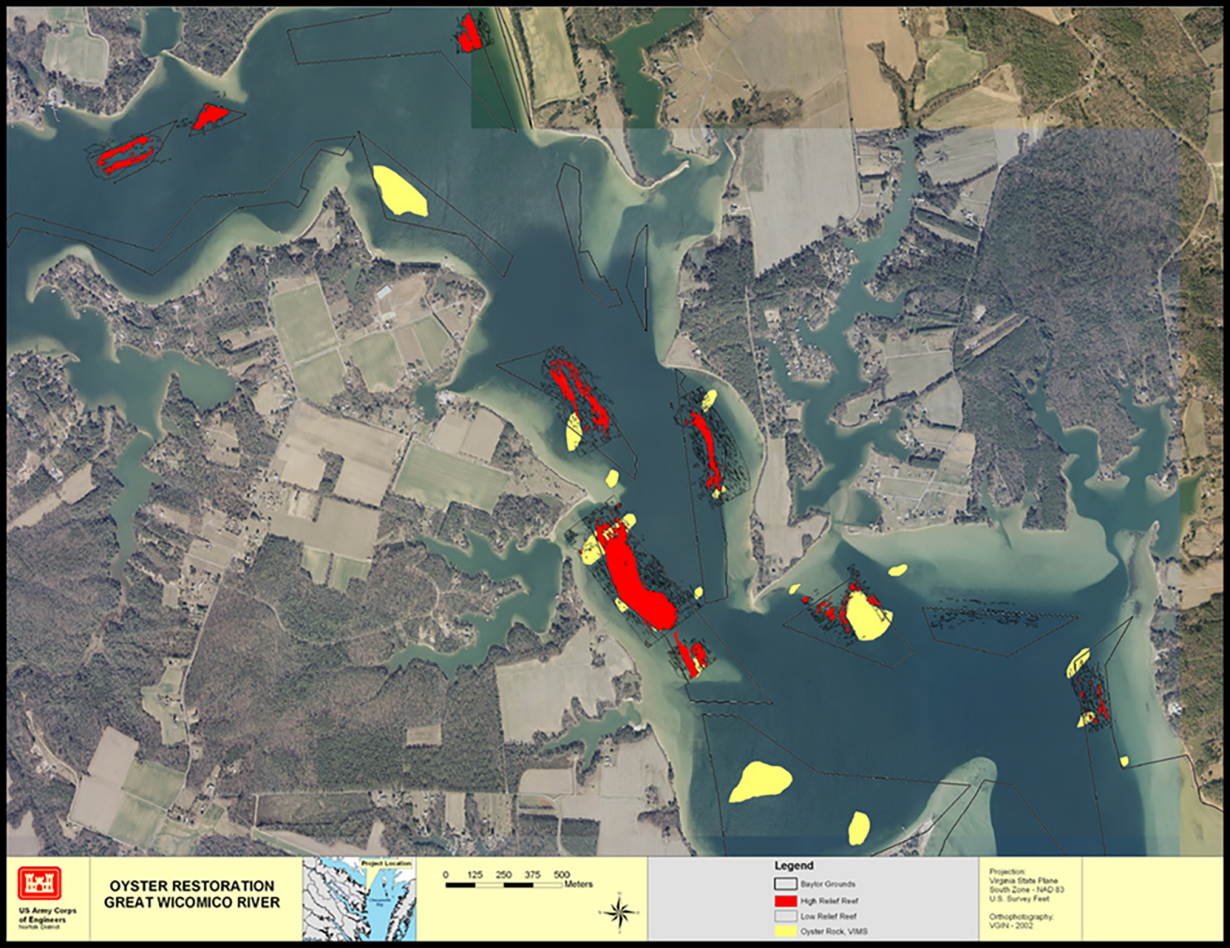
Great Wicomico River and restored oyster sanctuary reefs. From Schulte et al. 2009.

We used mechanically-operated patent tongs to survey an oyster population on a series of restored reefs in the Great Wicomico River (GWR), Chesapeake Bay, USA (Fig 3). These reefs were built in 2004 at varying heights on the bottom and separated into two categories, high relief (≥ 25 cm) and low relief (8–12 cm), and were the world’s largest oyster reef restoration project at the time. They were built out of shells dredged from formerly-productive oyster reefs now denuded of surface shell and covered by sediment. All reefs were built as sanctuaries and relied on natural recruitment to establish an oyster population on the reefs, which occurred (Schulte et al. 2009). Due to the world-wide [16], as well as Chesapeake Bay wide [17, 18] depletion of oysters and destruction of their habitat, oyster restoration efforts are expanding to restore this ecosystem engineer, which provides a wealth of ecological services [19, 20]. Due to significant and ongoing financial commitments in the Chesapeake Bay region towards oyster restoration, a goal implementation team (GIT) was formed in the Bay region, consisting of a mix of state and federal fishery managers, scientists, and those agencies (NOAA and the US Army Corps of Engineers) involved in construction and monitoring of restored oyster reefs. As it had been noted in the Chesapeake Bay scientific community that there is a lack of good monitoring data regarding oyster restoration efforts [21], we were keenly interested in sampling the GWR restoration project accurately and precisely such that results could be compared and contrasted with other restoration efforts as well as to evaluate the success of the reefs. We sampled in the winters of 2007–2008 and 2008–2009 using a device considered to produce very accurate results, a mechanically-operated patent tong with a measured area per grab of 1.03 m^2^. Tong size can vary, small tongs ranging from 0.1–0.25 m^2^ being used on smaller boats, which can access shallower reefs than those in the present study. The mechanically-operated patent tong relies on the weight of the device to penetrate the oyster reef and mechanical action to retrieve the sample. This is similar to the action of bottom samplers such as a PONAR grab, and its gear efficiency may be different from either SCUBA divers or the hydraulic patent tong.

While conducting the survey in winter 2007–2008, our patent tong samples were typically full, indicative of obtaining a complete sample. Additionally, there was a thin layer of whitish grey shells at the bottom of the sample, which were the original dredged shells used to construct the reef base of the sanctuary reefs, and were easily distinguishable from live oysters and newly-formed shell after reef construction. The dredged shells had been buried in anoxic sediments for several hundred years prior to their dredging and are distinct in physical appearance, as well as structurally, from new oyster shells [22], which are typically brown in color, longer and thinner.

When sampling reefs in the following year (winter 2008–2009), the patent tong did not appear to obtain a full sample consistently. Many samples, particularly those from high-relief reefs, were only partially full and the original dredged shell was absent from the bottom of the sample, suggesting that some of the live oysters were not collected. ROV video examination of sampled sites indicated that live oysters were being left behind within the sample site footprints. The reefs, especially the high-relief ones, appeared to have accreted substantial reef material, which could preclude the collection of complete samples with the patent tong within a single grab. Such was the case in the late 1800s, when Winslow (1882) sampled oyster reefs in Tangier Sound and Pocomoke Sound in the middle reaches of Chesapeake Bay. Winslow (1882) stated that obtaining accurate samples was much more difficult on high-density, cohesive, unfished beds compared to oyster beds that had been subject to dredging and tonging, due to the cohesiveness and much higher volume of oysters. Unfortunately, none of the earlier studies of oyster survey gear efficiency quantified the effect of reef characteristics on sampling efficiency [3, 10, 12–14]. Consequently, we aimed to assess the efficiency of mechanically-operated patent tongs in sampling subtidal oyster reefs constructed of shells, and determine if reef characteristics affected sampling efficiency.

We hypothesized that (1) hand-operated patent tong gear will be less than 100% efficient in sampling any oyster reef, (2) the patent tong will be more efficient in sampling low-relief reefs compared to high-relief reefs due to the cohesiveness of high-relief reefs, (3) the patent tong will sample adults more efficiently than young-of-the-year (YOY) oysters (= spat) due to the small size of the spat, and (4) processing of a sample will improve the sampling efficiency, due to subsampling selection, leading to a significantly greater survey efficiency than gear efficiency. Subsampling selection consisted of selecting the middle half of a grab, where a metal bar provides for a deeper grab for the middle half, while the two ends, which do not have as much weight to grab with, do not grab as deeply. We believe this will result in a higher efficiency rate for the middle half of the tong compared to either end.

## Methods

**Fig 4:**
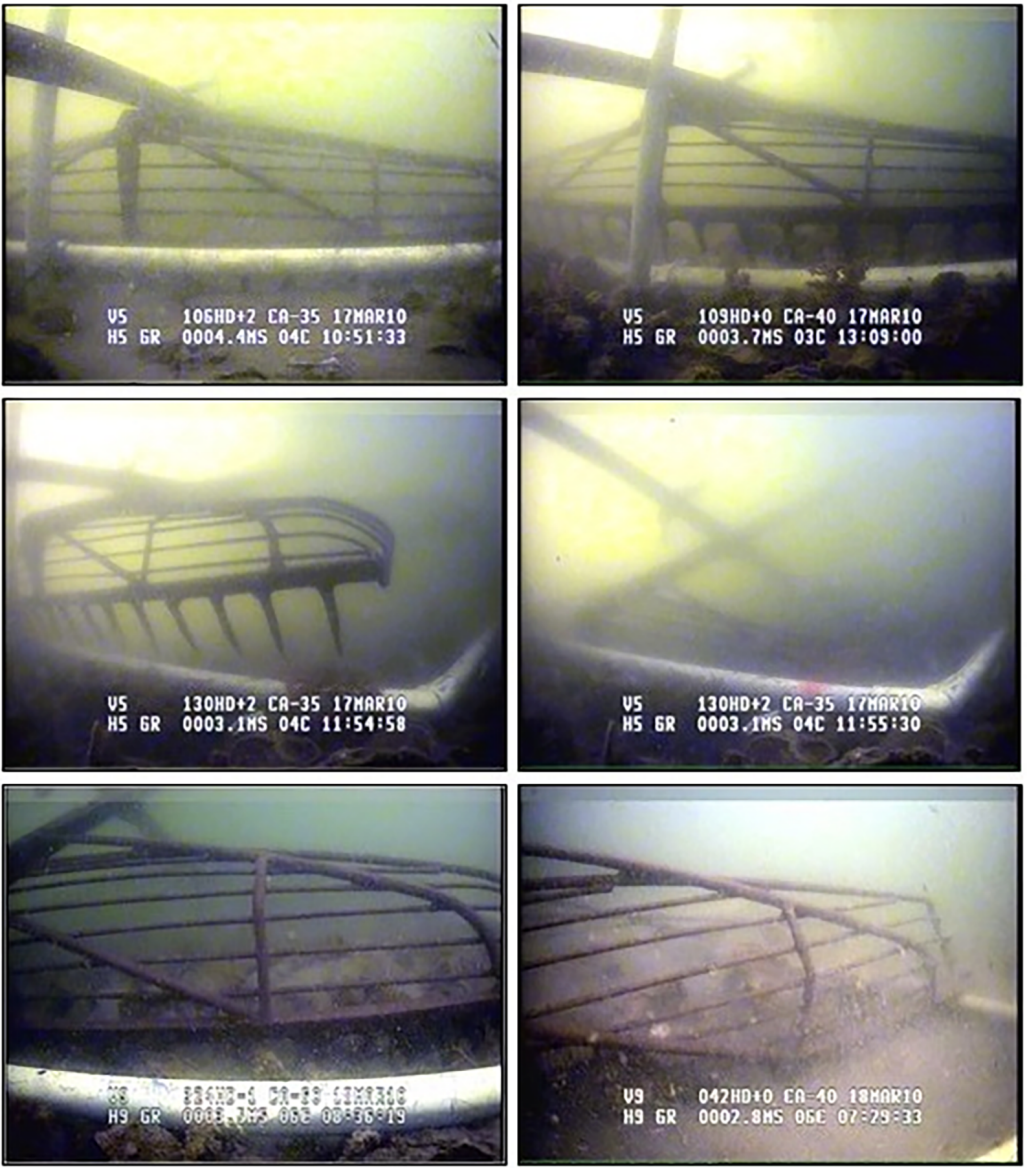
ROV images of patent tong and guiding device taking a reef sample.

**Fig 5:**
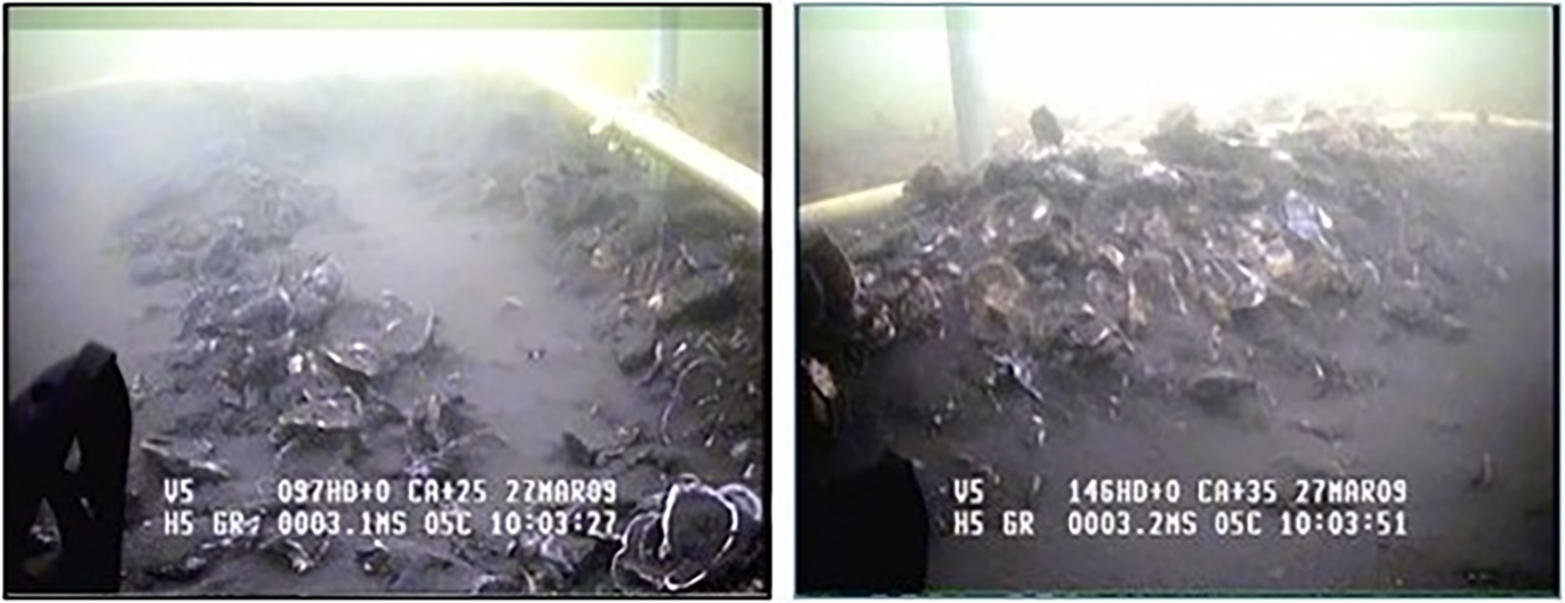
ROV images of sample area after 3 grabs, showing grey reef base shell.

Gear efficiency q (= catchability) relates the number (or biomass) of individuals caught in a sample (C) to the true number of individuals (N):

$$N=\frac{C}{q∙E}$$

where E = effort. In our survey we assume E = 1 because we are interested in estimating the efficiency of a 1-m2 sample. Survey efficiency is a product of availability (a), gear efficiency (g), and processing efficiency (p):

q=a∙p∙g

although most surveys assume processing efficiency = 1 and, therefore, do not include it in calculations [23]. We also assume that a = 1 because the oyster reefs were defined by side-scan sonar mapping, oysters are immobile, and our sampling grids encompassed the whole of each reef. To estimate gear efficiency, we developed a device consisting of a metal frame designed to guide the patent tong to repeatedly grab in the same sample area until no live oysters were in the sample (Fig 4). An ROV was used to verify that there were no more live oysters in the plot, that the patent tong was placed within the same sampling area for multiple grabs, and that no additional material from outside the sampled area was captured in the grabs. Fig 4 illustrates the guide and tong while taking repeated grabs and the appearance of a sampled area denuded of all live oysters (100% of the sample gathered). Typically, this took 2–3 grabs to achieve. By the end of the third grab, a hole approximately 10–15 cm deep was evident, denuded of live oysters with only the original, grey “fossil” shell used to build the reefs, and bottom sediments were also evident (Fig 5). Gear efficiency was estimated with a series of grabs during winter surveys of the restored GWR oyster reefs in 2008–09 and 2009–2010. These reefs consisted of a network of 8 reefs that had both high- and low-relief habitat within the restoration polygon. A ninth reef, the one immediately to the southwest of the largest restored reef, had been built using different methods years before, and was not part of our experiment (Fig 3). Both high- and low-relief reefs were sampled (n = 11 for high, and n = 8 for low) to test our first two hypotheses. Due to the difficulty of obtaining samples, with a single sample taking approximately 4 h, we sampled a randomly selected subset of the restoration reefs each year, rather than from all reefs. Two of the reefs were not sampled during our experiment. As reef type was considered a stratum (high and low) during prior work [7] we sampled the reefs by stratum in the present study, which did not require a specific number of samples from each individual reef. As we were trying to estimate the efficiency of the tong by strata, this is appropriate. Each reef was divided into a grid of numbered 10 m^2^ blocks, and a random number generator was used to select blocks to sample. Significantly more oysters were found on the high-relief strata (463 ± 47.55 SE, n = 11) than the low-relief strata (211.5 ± 62.21 SE, n = 8). All data, including the locations of sampling points, are in S1 and S2 Tables.

To estimate survey efficiency, we followed the same methodology used in our surveys. Specifically, we divided the sample into two halves, one of which we kept for processing while the other was returned overboard as close as possible to the original sampling location. For the processed half, we selected the half over the metal bar in the middle of the patent tong. The tong's heavier weight in the middle due to the metal bar provides for a deeper grab compared to either end, likely improving efficiency. Our unselected half of the sample was the two ends of the sample where the tong has less weight and a shallower grab. Samples were taken back to the lab for processing, and the data multiplied by two to produce an estimate per m^2^, the full grab area. These estimates were then divided by the total number of oysters in each full grab, as described above, to generate survey efficiency. As we are hypothesizing that this method will produce a higher efficiency rate, no bias is introduced into the data relative to our expected lower efficiency of the gear only. A lower efficiency rate for gear would produce a larger multiplier to obtain the total number of oysters, including those missed in the first grab, relative to the survey efficiency rate. Both efficiencies should thereby produce a similar result, though if the estimates of gear and survey efficiency differ radically from each other, one method may be more accurate than the other.

To address our hypotheses regarding the efficiency of the patent tong, we developed statistical models with various combinations of fixed factors including reef relief (high or low), oyster size (spat or adults), and year (2008–2009 or 2009–2010), and total oyster density summed across all grabs in a plot as a covariate (Tables 1 and 2). Spat were ≤ 35 mm shell length (= shell height) [3, 7], while adults were > 35 mm shell length. Generalized linear models (GLM) with a Gaussian distribution (*y =* β_0_ + β_1_x_1_ +…+ β_p_x_p_ + *e*) were run separately for gear efficiency and survey efficiency data. Statistical analyses were conducted using R [24] and RStudio [25] statistical software.

**Table 1:** Akaike information criterion analysis for the gear efficiency rate. K is the number of estimated parameters in the model; AIC_c_ is the second order (due to small sample size) Akaike’s information criterion value; Δ_i_ is the delta AIC, which is the difference between each model and the best model; and *w*_*i*_ is the Akaike weight, which indicates the probability that the model is the best among the candidate models.

Model	Variables	k	AIC_C_	Δ_i_	*w*_*i*_
*g*_*1*_	Oysters*Relief*Year	9	257.77	28.38	<0.01
*g*_*2*_	Oysters+Relief+Year	5	236.32	6.93	0.019
*g*_*3*_	Oysters+Relief	4	232.56	3.17	0.129
*g*_*4*_	Oysters*Relief	5	236.14	6.75	0.043
*g*_*5*_	Oysters+Year	4	232.63	3.24	0.124
*g*_*6*_	Oysters*Year	5	235.66	6.27	0.056
*g*_*7*_	Oysters	3	229.39	0	0.629
*g*_*8*_	Null	2	256.47	27.08	<0.01

**Table 2:** Akaike information criterion analysis for the survey efficiency rate. K is the number of estimated parameters in the model; AIC_c_ is the second order (due to small sample size) Akaike’s information criterion value; Δ_i_ is the delta AIC, which is the difference between each model and the best model; and *w*_*i*_ is the Akaike weight, which indicates the probability that the model is the best among the candidate models.

Model	Variables	k	AIC_C_	Δ_i_	*w*_*i*_
*g*_*1*_	Oysters*Relief*Year	9	232.33	26.33	<0.01
*g*_*2*_	Oysters+Relief+Year	5	212.91	6.91	0.021
*g*_*3*_	Oysters+Relief	4	209.17	3.17	0.136
*g*_*4*_	Oysters*Relief	5	211.38	5.38	0.022
*g*_*5*_	Oysters+Year	4	209.25	3.25	0.131
*g*_*6*_	Oysters*Year	5	210.85	4.85	0.029
*g*_*7*_	Oysters	3	205.99	0	0.661
*g*_*8*_	Null	2	251.13	45.12	<0.01

We used an information-theoretic approach [26, 27] to select the best-fitting statistical model(s) from a set of eight models (*g*_*1*_*-g*_*8*_), including the null model for comparison (Tables 1 and 2). Catch from each sample was modeled as a continuous response. Each model was analyzed using the bias-corrected Akaike Information Criterion (AIC_C_). Model probabilities (*w*_*i*_) based on Δ_i_ values were used to determine the probability that a particular model was the best-fitting model. Chi square (X^2^) tests were used to assess the fit of the best model relative to other models, including the null model. The two efficiency rates (gear and survey) were compared with a one-tailed, paired t-test to determine if survey efficiency was significantly higher than gear efficiency.

## Results

**Fig 6:**
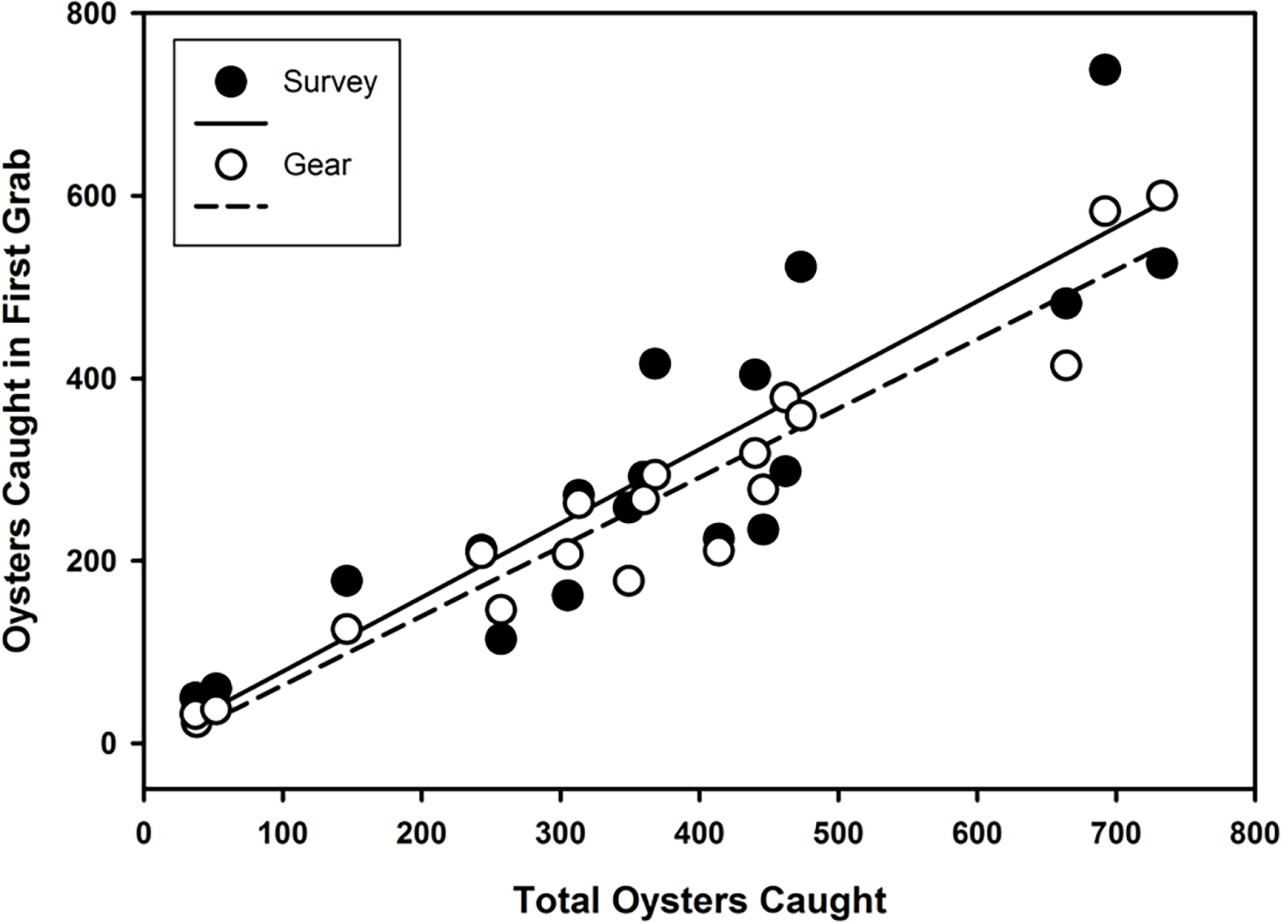
Survey efficiency (*y = -2*.*280+0*.*811x*, *r*^*2*^ *= 0*.*79*) and gear efficiency (*y = -12*.*373+0*.*759x*, *r*^*2*^ *= 0*.*92*), which is the first grab vs. the total number of oysters in a sampling plot caught using multiple grabs.

Catchability of adults and spat did not differ significantly for either the gear (F = 0.290 df = 7, 30, *p* = 0.953) or survey (F = 1.243, df = 7, 30, *p* = 0.311) efficiency. Spat and adults were subsequently added together and analyzed as total oyster abundance. For gear efficiency, model *g*_*7*_ (oyster abundance only) had the highest *w*_*i*_, 0.629, with the next best fitting models being *g*_*5*_ (*w*_*i*_ = 0.124) and *g*_*3*_ (*w*_*i*_ = 0.129) (Table 1). Models *g*_*5*_ and *g*_*3*_ were eliminated from further consideration because their parameter estimates were not significant (Table 3). Similarly, for survey efficiency, model *g*_7_ also had the highest *w*_*i*_, 0.661, with the next best fitting models being *g*_*5*_ (*w*_*i*_ = 0.131) and *g*_*3*_ (*w*_*i*_ = 0.136) (Table 2). For both efficiency rates, model *g*_*7*_ provided a significantly better fit than all other models (X^2^ test, *p* < 0.001), and explained a significant fraction of the variance (Fig 6).

**Table 3:** Parameter estimates from best-fitting generalized linear models with Gaussian distribution.

Model	Parameter	Variable	Estimate	SE	t-value	p
			*Gear*			
*g*_*7*_	β_0_	Intercept	-12.3725	22.252	-0.556	0.585
	Β_1_	Oysters	0.7593	0.05432	13.977	<0.001
*g*_*5*_	β_0_	Intercept	-11.1070	35.8836	-0.31	0.761
	β_1_	Oysters	0.7574	0.06912	10.959	<0.001
	β_2_	Year	-1.2695	27.683	-0.046	0.964
*g*_*3*_	β_0_	Intercept	-19.9889	36.2071	-0.552	0.589
	β_1_	Oysters	0.7714	0.07138	10.806	<0.001
* *	β_2_	Relief	7.8486	28.9144	0.271	0.79
			*Survey*			
*g*_*7*_	β_0_	Intercept	-2.2800	41.1842	-0.055	0.956
	β_1_	Oysters	0.8114	0.1005	8.071	<0.001
*g*_*5*_	β_0_	Intercept	3.9945	66.3868	0.06	0.953
	β_1_	Oysters	0.8022	0.1279	6.274	<0.001
	β_2_	Year	-6.2940	51.2151	-0.123	0.904
*g*_*3*_	β_0_	Intercept	-16.3992	67.0119	-0.245	0.81
	β_1_	Oysters	0.8338	0.1321	6.311	<0.001
* *	β_2_	Relief	14.5500	53.5147	0.272	0.789

For model *g*_*7*_, the gear efficiency rate was estimated at 76% ± 5% (standard error) and the survey efficiency at 81% ± 10% (Fig 6, Table 3). Survey efficiency was significantly higher than gear efficiency (one-tailed, t = 1.774, df = 18, *p* = 0.048).

## Discussion

We hypothesized that (1) hand-operated patent tong gear will be less than 100% efficient in sampling any oyster reef, (2) the patent tong will be more efficient in sampling low-relief reefs compared to high-relief reefs due to the cohesiveness of high-relief reefs, (3) the patent tong will sample adults more efficiently than young-of-the-year (YOY) oysters (= spat) due to the small size of the spat, and (4) processing of a sample will improve the sampling efficiency, due to subsampling selection, leading to a significantly greater survey efficiency than gear efficiency. Our results support hypotheses 1 and 4; 2 and 3 were rejected. Hypothesis 1 was supported because gear efficiency of the mechanically-operated patent tong was 76% (± 5% standard error) for the first grab, not 100% efficient in capturing all live oysters in a single grab. In contrast, gear efficiency of hydraulic patent tongs is assumed to be 100% because efficiency of the hydraulic patent tong did not differ significantly from that of SCUBA divers, who were instructed to excavate all oysters and associated reef material down to 10 cm below the surface of the reef [3]. Unfortunately, the precision of the density estimates in [3] was very low, which could have precluded detection of a difference. In addition, processing efficiency was not estimated, and densities were extremely low (2–35 oysters m^-2^), such that the results are not necessarily applicable to the much higher densities on restoration oyster reefs [7, 8]. If hydraulic tongs are in fact more efficient than mechanical tongs, it is likely due to the greater weight of the device and, more importantly, its closure under power which allows it to actively dig into the reef substrate. The mechanically-operated patent tong is likely less than 100% efficient due to its lighter weight and unpowered retrieval of the sample as the tong is raised. Although we observed that the mechanical tong digs into the substrate during retrieval, it relies only on the weight of the device to do so.

An important element of our study was inclusion of processing efficiency to estimate survey efficiency separately from gear efficiency on the first grab, which no other study has done to our knowledge. Processing can affect survey efficiency either positively or negatively. In our case, processing positively raised survey efficiency from 76% (gear alone) to 81% (gear + processing), supporting hypothesis 4. This was likely due to our preferential selection of the middle half of the sample, which maintained its structural integrity better and appeared to dig deeper into the reef structure when compared to the ends of the grab. For oyster surveys that use a mechanically operated patent tong, we suggest the gear efficiency rate is 76% for the full first grab, but if the middle half of the sample is selected for further assessment, the efficiency rate is 81%. In both cases, only a single grab per sample point is necessary. The r^2^ was higher (0.92) for gear than for survey (0.79) efficiency, indicating that assessing the full sample will likely produce a more accurate density estimate and a smaller standard error. However, the survey samples, which are half the volume of the gear samples, require much less lab space and processing time to assess.

Gear and survey efficiency did not differ significantly between high- and low-relief reefs, causing us to reject hypothesis 2. The reefs we sampled were much younger than those sampled in the era of Winslow (1882), three to five years, instead of centuries, old. They may simply not hold enough generations of oysters fixed together into a cohesive structure that would negatively influence the efficiency of the patent tong. The more contracted size-frequency distributions on modern reefs due to disease and harvesting may also be easier to sample when compared to historic reefs [18, 28–32] that held more age classes with larger adults, as well as greater cohesion and rugosity. It may take many generations of oysters on an unfished sanctuary reef to produce a truly cohesive reef similar to unexploited reefs found in the Winslow era. Oyster density could also be a factor. Reefs examined in the Chai et al. (1992) study held very low numbers of oysters (2, 18 and 35 m^-2^ on their three reefs), so most of the reefs in that study consisted of empty shell. Catchability was likely higher for the few adult oysters present, as they were not part of a cohesive structure but simply sat on top of loose shell, which was easily grabbed by a patent tong of either design. Small spat on loose shells may be easier to miss in such samples, especially if counting is done in the field under less-than-ideal weather conditions. If any difference in efficiency between high- and low-relief reefs had been identified in our study, it would likely have been due to differences in the population demographics, as well as rugosity and cohesiveness between reef types [7, 15, 31–36] but this was not observed on these young (constructed in 2004) reefs. If left undisturbed as sanctuaries over a multi-decadal timeframe, it is probable that such differences will be noted.

Regarding our rejection of hypothesis 3, larvae preferentially settle on shells of live adults [37] embedded in the reef, so most spat are fixed to either a live adult oyster or a large oyster shell near an adult so it is not unexpected that the catchability of spat is similar to that of the adults. We found that poor weather in the field (reefs are typically sampled in the winter, where temperatures often drop below 5°C) does tend to result in less care taken processing a sample. In a sample with over 1,000 oysters, including many spat less than 5 mm shell length, counting in the lab compared to counting in the field ensures accuracy. Catchability between age classes was examined using an oyster dredge [15] who also found no significant difference between size classes. This is often the case for other gear-based molluscan fisheries, such as that for the European surf clam *Spisula solida* [38] and Venus clam *Anomalocardia brasiliana* [39], where the fishing gear, in both cases a metal-toothed dredge, was not size selective via the frame of the dredge. It is expected that the frame of the metal-toothed patent tong is similar. When the mollusk is motile, unlike oysters, size selectivity can occur, but this can be due to, such as in the case of young (< 100 mm) sea scallops (*Placopecten magellanicus*), to the ability of juveniles to swim above the dredge, unlike larger adults [40].

We developed the survey method as a cost and space-savings measure, as only half the sample required transport back to the lab, storage, and processing. Our study demonstrated that the mechanically-operated patent tong is not 100% efficient in catchabilty on its first grab at 76%, which can be increased by our survey method of only taking the middle half of the sample to 81%. A disadvantage of the survey method vs the gear method, where the entire sample is assessed, is a small sacrifice in model fit as the r^2^ is slightly higher (0.92 compared to 0.79). If time, funding, lab space and personnel permit, assessing the full sample will provide a better fit to the data, though the survey method still provides a very good data fit. Our method using the metal guide allowed for multiple grabs within the same sample point which allowed us to obtain all the live oysters in a particular sample area. While this level of accuracy is desirable, the time in the field was excessive, as we could typically obtain only 2 samples per day as carefully guiding the tong into the exact spot using the ROV and metal guide was a tedious process on a boat, even anchored, in moving tidal waters with wave activity. We recommend taking only one grab per sample site and using the appropriate efficiency rate. This also has the benefit of reducing the damage to the reef by sampling, which is not insignificant and should be minimized to the fullest extent practicable. Regarding this damage due to sampling, this is one of the reasons we selected the patent tong over the dredge, in addition to the inaccuracy of the dredge. Dredges, which are scraped over wide areas of a reef, significantly damage oysters and reef structure along the entire transect, which can measure over 100 m in length, destroying any cohesion, knocking clusters over and killing oysters by either hitting and damaging their shells or altering their position on the reef such that they cannot feed or breathe. Scuba and tonging damage a reef much less, only leaving discrete small pits on the reef surface, approximately 1 m^2^ (or less) in size. While much less damaging than a dredge, this still impacts the reef as such pits are likely to fill with sediment unless the oysters can quickly recover and achieve vertical growth within the depression.

Financial costs should also be considered. From our experience, a pair of divers and associated boat and fuel expenses were approximately US$1,000 per day, while expenses for a patent tong boat and fuel were approximately US$500 per day. Divers are best in waters < 2 m deep, as a tong boat cannot effectively navigate in less than 1.5 m deep water. Tongs are more effective in deeper waters, able to retrieve more samples per unit time than divers and making them even more cost-effective in this situation. We recommend divers in shallow waters and to sample alternative material reefs, but recommend patent tongs in deeper waters on shell or small stone (3–12 cm diameter) reefs.

Possible limitations of the mechanical tong as a survey method could be lower efficiency at low (< 25 oysters m^-2^) densities, as it is likely easier to miss small numbers of oysters on poor quality reefs with no cohesion or clusters, both of which provide a greater chance of being grabbed by the device due to greater size and rugosity. It is also possible that cohesive, very high density reefs may be more difficult to sample with lower efficiency. While we suspected this might be the case for the high-relief reefs due to their greater densities and cohesiveness, this did not prove to be the case. On natural, unexploited reefs with even higher densities than found on the high-relief reefs in the present study, however, we suspect efficiency will decline due to difficulty in penetrating the reef. Another limitation of the device is the inability to sample bioengineered reef constructed out of alternative materials, such as reef balls and other shaped objects, where divers or ROV must still be used.

## Supporting information

S1 Table Gear and survey data. (DOCX)

S2 Table Survey point locations. (DOCX)
