# Supplementary material for: Gear and survey efficiency of patent tongs for oyster populations on restoration reefs
Source: PLoS One. 2018 May 2;13(5):e0196725. doi: 10.1371/journal.pone.0196725 (PMC5931685; doi:10.1371/journal.pone.0196725)
Supplement: S1 Table — (DOCX) [file pone.0196725.s001.docx]

**S1 Table. Gear and survey data.**
