# Supplementary material for: Gear and survey efficiency of patent tongs for oyster populations on restoration reefs
Source: PLoS One. 2018 May 2;13(5):e0196725. doi: 10.1371/journal.pone.0196725 (PMC5931685; doi:10.1371/journal.pone.0196725)
Supplement: S2 Table — (DOCX) [file pone.0196725.s002.docx]

**S2 Table. Survey point locations.**

| **Relief** | **Year** | **Reef** | **Site** | **Latitude** | **Longitude** |
| --- | --- | --- | --- | --- | --- |
| HRR | 2008 | 11 | 163 | 37.83052 | -76.3204 |
| HRR | 2008 | 4 | 129 | 37.85338 | -76.32868 |
| HRR | 2008 | 11 | 97 | 37.83027 | -76.31972 |
| HRR | 2008 | 16 | 121 | 37.8264 | -76.298 |
| HRR | 2008 | 16 | 254 | 37.82747 | -76.29847 |
| HRR | 2008 | 9 | 131 | 37.83615 | -76.31677 |
| LRR | 2008 | 8 | 212 | 37.83835 | -76.32317 |
| LRR | 2008 | 1 | 127 | 37.84867 | -76.34563 |
| LRR | 2008 | 9 | 98 | 37.8358 | -76.3162 |
| LRR | 2008 | 9 | 221 | 37.83705 | -76.31645 |
| HRR | 2009 | 11 | 435 | 37.8316 | -76.32143 |
| HRR | 2009 | 16 | 155 | 37.82666 | -76.29826 |
| HRR | 2009 | 10 | 23 | 37.8328 | -76.32143 |
| HRR | 2009 | 16 | 218 | 37.82718 | -76.29876 |
| HRR | 2009 | 4 | 39 | 37.85256 | -76.32787 |
| LRR | 2009 | 2 | 85 | 37.84922 | -76.3441 |
| LRR | 2009 | 2 | 65 | 37.84953 | -76.34354 |
| LRR | 2009 | 10 | 223 | 37.8341 | -76.32177 |
| LRR | 2009 | 1 | 59 | 37.84835 | -76.34626 |
